# The Role of IOS in Identification of Specific Treatable Traits in Pediatric Asthma: Current Limitations and Future Perspectives—Narrative Review

**DOI:** 10.3390/jcm14207368

**Published:** 2025-10-18

**Authors:** Joanna Połomska, Hanna Sikorska-Szaflik, Barbara Sozańska

**Affiliations:** Department and Clinic of Paediatrics, Allergology and Cardiology, Wroclaw Medical University, Ul. Chałubińskiego 2a, 50-368 Wrocław, Poland; hanna.sikorska-szaflik@umw.edu.pl (H.S.-S.); barbara.sozanska@umw.edu.pl (B.S.)

**Keywords:** pediatric asthma, oscillometry, small airway disease, treatable traits, diagnostic advances

## Abstract

Asthma management in children aims to prevent ongoing symptoms, preserve lung function and support normal daily activities. Impulse oscillometry (IOS) represents a modern approach to evaluating lung function that is also suitable for performing in the pediatric asthma population. Further research is warranted to clarify the role of IOS in the early identification of small airway disease (SAD) as a potential treatable asthma trait and to understand its implications for personalized treatment strategies. Before the integration of IOS into routine clinical protocols, it is necessary to establish population-specific reference values. Further studies in the pediatric population are needed to evaluate the added value of IOS in combination with conventional spirometry and fractional exhaled nitric oxide (FeNO). Future pediatric asthma management guidelines may consider incorporating the assessment of SAD with IOS as a possible tool for its evaluation.

## 1. Introduction

Asthma management may be characterized as a persistent process of evaluation and reassessment in areas such as clinical symptoms, modifiable and non-modifiable risk factors, co-existing conditions, adverse effects of treatment and quality of life [1]. The clinical presentation of asthma in the pediatric population is often challenging to assess objectively. Pediatric asthma often has its onset in early childhood, and approximately 50% of individuals experience wheezing by six years of age [2]. Current diagnostic tools, including spirometry, are limited for evaluating pulmonary function in preschool-aged children and the required respiratory effort precludes reliable testing during episodes of flare-ups, commonly triggered by viral infections in this population. A recent systematic review and meta-analysis demonstrated that severe asthma may affect approximately 3% of pediatric asthma patients in Europe; however, the underlying mechanisms in the pediatric population remain insufficiently investigated [3].

Children with severe asthma may demonstrate normal spirometric values or only mild obstruction when not experiencing an acute exacerbation [4]. Therefore, there is a growing need to implement an additional non-invasive functional lung test offering support in asthma diagnosis in early childhood, sensitive identification of the onset of exacerbation and suitability for repeated use during acute asthma episodes. Nowadays, impulse oscillometry (IOS) is proposed as a complementary test to routine spirometry—unlike spirometry, IOS can assess the peripheral airways, as airflow limitation in asthma predominantly occurs in small airways. Small airway disease (SAD) is now considered a potential reason why some patients, despite a confirmed diagnosis and anti-inflammatory therapy, continue to exhibit uncontrolled asthma. It is increasingly recognized as a treatable asthma trait, offering new possibilities for targeted intervention [5].

This narrative review discusses the application of IOS in asthma management in the pediatric population, moving the theoretical concepts to practice, and is based on a literature search in PubMed, Google Scholar and Web of Science using the keywords ‘pediatric asthma’, ‘oscillometry’, ‘small airway disease’ and ‘treatable traits’, focusing on publications from 1989 to 2025, with the majority published within the last decade. Nevertheless, the findings of this review should be interpreted with consideration of the variability in study designs, populations and methodologies, which may influence the extent of generalizability. Furthermore, the review was limited to studies indexed in selected databases, which might have resulted in the omission of some relevant literature. In this review, we also highlight the key advantages and limitations of IOS, together with the implementation issues that restrict its regular use in clinical practice with children

## 2. Impulse Oscillometry

### 2.1. Overview and Clinical Relevance

IOS, a variant of the forced oscillation technique, is a type of non-invasive and patient-friendly lung function test that requires only tidal breathing instead of forced and complicated breathing maneuvers performed for spirometry. This technique is applicable in preschool-aged children with asthma without altering their prevailing breathing pattern and without affecting airway geometry [5,6]. Even though this method seems to be technically easier than spirometry, it should not be regarded as a substitute for spirometry nor as a pulmonary function test only useful in early childhood patients. Basically, it would be inappropriate to consider IOS as a diagnostic method to use until the pediatric patient is cooperative enough to properly perform spirometry. IOS remains relevant also as the asthmatic grows up. In addition, spirometry cannot serve as a reference point for the results of IOS measurements as these two diagnostic tools measure entirely different parameters. It is possible to use this technique together with other assessments, e.g., spirometry, fractional exhaled nitric oxide (FeNO), plethysmography and other measurements in specialized pulmonary units. Nowadays, asthma management focuses on the combination of these techniques that seem to complement, not replace, each other [7]. IOS is recommended when asthma symptoms are poorly controlled despite normal spirometry results. In this clinical context, SAD should be considered among other factors that may hinder the attainment of optimal treatment outcomes.

### 2.2. Technical Aspects of IOS

The “Technical standards for respiratory oscillometry” standard was published by the European Respiratory Society (ERS) in 2020 to summarize the current state of knowledge regarding equipment, software, the diagnostic procedure and quality control. IOS is performed before other diagnostic methods requiring forced or prolonged breathing. During the examination, patients sit upright with a nose clip, breathe through a special mouthpiece and stabilize their cheeks [8,9]. There is no need to coordinate the respiratory phases with any special breathing maneuvers, although patients must stay calm and focus on normal breathing. The waves are generated by a loudspeaker, which is an integral part of the device, and then the waves are superimposed onto the patient’s spontaneous physiologic breathing. The test is not time-consuming, as it consists of at least three measurements lasting 20–35 s of normal breathing and unreliable assessment may be repeated [10].

While considered easy to perform, IOS requires some patient cooperation. Correct height input is necessary, because height is considered to be the most influential predictor of respiratory resistance [10]. Data on the influence of sex on IOS parameters in children across different age groups are inconsistent. Table 1 lists examples of methodological limitations and artifacts affecting result accuracy [8,11,12,13].

### 2.3. IOS Parameters and Interpretation

The conducted measurement provides the oscillogram depicting oscillometric impedance (Zrs) expressed in kilopascal per liter per second [kPa/L/s] and is represented by its components, including resistance (Rrs) and reactance (Xrs), plotted against frequency. Rrs is defined as the in-phase part of respiratory impedance and depends on the bronchial cross-section radius and the bronchial length, and expresses mainly the friction to airflow. Rrs5 stands for resistance measured at a frequency of 5 Hertz (Hz), and provides data on total airway resistance, whereas Rrs20 stands for resistance measured at a frequency of 20 Hz, reflecting proximal airway resistance. Therefore, Rrs5–20 means subtraction of Rrs20 from Rrs5 and is used to define the central or peripheral location of obstruction. Xrs is described as the out-of-phase respiratory impedance and reflects elasticity (including chest wall and bronchi walls, lung parenchyma) and inertia of gas in the respiratory system. Ventilation inhomogeneity and lung stiffness are expressed as elevated Xrs or area under the reactance curve (AUC) [8].

### 2.4. Clinical Relevance and Limitations

The interest in IOS is constantly growing, especially due to recent task force reports indicating IOS as a useful lung function test in asthma management. The significance and application of this method were described in the *European Respiratory Review* in 2022. The authors provided a concise overview of the physiological basis of IOS and key aspects of interpretation. Their review highlights that IOS may be advantageous in situations where standard lung function tests are not feasible or provide limited reliability (infants, individuals with neuromuscular disorders, patients with sleep apnea or those in critical care settings). It also points to additional potential applications including bronchitis obliterans, vocal cord dysfunction and the assessment of respiratory effects from environmental exposures. Current evidence supporting IOS as a sensitive method for assessing airway resistance, bronchodilator response, bronchial challenge and treatment effects in asthma and chronic obstructive pulmonary disease was summarized.

However, IOS is still not widely used in managing asthma. Unfortunately, simplicity in performing IOS measurement does not correspond to simplicity in result interpretation and incorporation into therapeutic decision-making. The latest publications indicate the unrecognized value of IOS as well as research questions that still need to be answered [14]. Nowadays, there is an idea to collect respiratory function outcomes and prepare universal reference quotations for IOS measurements by the Global Lung Function Initiative. In the future, large-size multicenter and multinational studies including both pediatric and adult populations are necessary to provide normal reference values. These data need to be thoroughly processed before IOS can take its rightful place among established diagnostic approaches to asthma.

## 3. Small Airway Disease

### 3.1. Overview

In routine clinical practice, spirometry, as a reliable, robust clinical tool, is still a method of choice in assessing lung function universally recommended for asthma management, whereas IOS, because of a lack of globally accepted reference values, remains rather a research tool [15]. Forced expiratory volume in one second (FEV1) is suitable to evaluate central airways and is indicative of the reversible or variable nature of bronchial obstruction, but not peripheral airways, and is less sensitive than IOS in the detection of SAD. The mid-expiratory flow rate between 25% and 75% of forced vital capacity represents SAD in spirometry, but this is not an ideal method, because the outcomes depend on the lung volume parameters [16].

In anatomical terms, SAD refers to impairment ranging from the 8th to 23rd generation of the bronchial tree including acinar airways and conducting airways of internal diameter less than 2 mm. From the pathopsychological point of view, small airways may be the main or the only site of airway obstruction with a narrowed internal lumen caused by chronic inflammation, infiltration of eosinophiles and other immune cells, thickening of the smooth muscle layer and accumulation of mucous secretion [15,17]. Clinical consequences include more severe asthmatic symptoms and greater exercise-induced airway hyperresponsiveness or bronchial hyperreactivity triggered by allergen exposure, as well as increased frequency of asthma exacerbations in long-term observations [18]. Small airways have a total surface area of approximately 300 square centimeters throughout the alveolar region, and persistent inflammation of these peripheral parts of the lung contributes to suboptimal asthma control [19].

### 3.2. SAD and Asthma Control

Results of recent studies highlight that persistent inflammation and peripheral airway dysfunction are among the risk factors for persistent asthma and loss of lung function with age. SAD correlates with asthma severity stage and the concept of SAD at every stage of asthma makes the instrumental identification and assessment of their impairment in children extremely necessary [20]. The overview of research indicates that IOS would be useful in the classification of SAD as it correlates to the level of type 2 inflammation and different degrees of airway obstruction [21].

Currently, attempts are being made to characterize SAD phenotype including demographic features and clinical manifestations, loss in respiratory function, inflammatory markers and other data such as pH of alveolar breath condensate [22]. It is necessary that the results of lung function tests reflect the clinical manifestation of SAD. IOS is intended to be suitable to assess both small and large airways with possible distinction between peripheral and central obstruction [23]. To capture the nature of SAD in asthmatic children, extensive research should be performed. There is no clear understanding of the differences in loss of lung function following SAD between asthmatic children and adults. The outcomes of current studies on adult asthmatics cannot be extrapolated to asthmatic children.

### 3.3. Treatable Asthma Traits and SAD

Recommendations emphasize the importance of recognizing asthma phenotypes/endotypes and treatable traits. The concept of treatable asthma traits has been proposed as a strategy to recognize and modify pulmonary, extrapulmonary and behavioral factors that affect asthma control [24]. So far, several well-defined pulmonary asthma treatable traits are indicated, such as airflow limitation and airway inflammation, and SAD is currently regarded as another candidate among them [21]. Ascertainment of SAD as a hallmark of asthma could lead to better symptom control [25]. Children with uncontrolled asthma are at higher risk of asthma exacerbations, use of systemic corticosteroids and loss of lung function. Therefore, it is clear that good asthma control becomes a main goal [26].

## 4. IOS in Diagnosis and Therapeutic Management of SAD

### 4.1. IOS in Adults

Cottini et al. conducted studies on IOS-defined SAD in a population of asthmatic patients with normal spirometry. In addition, the study evaluated SAD prevalence in the study population based on the spirometry measurements interpreted as forced expiratory flow between 25% and 75% of vital capacity (FEF 25–75) less than 65% of predicted values. It confirmed that IOS measurements in asthmatics with preserved spirometry may increase the sensitivity of SAD detection [27].

A specific strategy to investigate SAD based on the combination of tests has been proposed in different asthma populations, which encompasses reduced FEF 25–75 (spirometry) together with other techniques such as lung volume measurement (plethysmography), resistance assessment (IOS) and inflammation evaluation (FeNO). The most comprehensive study of this kind was conducted by Postma and her research team between 2014 and 2017. In a multinational prospective cohort study, the Assessment of Small Airways Involvement in Asthma (Atlantis Study), participants aged 18–65 years with asthma and healthy controls were recruited to evaluate the presence of SAD using the panel of tests. The authors demonstrated that the prevalence of SAD was identified with differing frequency in the studied population depending on the method of assessment. Results of the multiple breath nitrogen washout were associated with the lowest prevalence of SAD, whereas IOS and spirometric outcomes reflect the clinical SAD scores [7].

Takeda et al. used IOS, spirometry and questionnaire-based assessment to investigate the potential association between SAD, clinical symptoms and disease control. A study was conducted on a group of 65 adult patients with no history of asthma exacerbation in the past four weeks. The Japanese version of the Baseline Dyspnea Index was used to assess dyspnea in asthma. It was demonstrated that in IOS measurements, small airways obstruction was correlated with increased airway resistance, mainly in the lower frequency range. The study found that asthmatic symptoms such as episodes of wheezing, dyspnea and chest tightness were markedly linked to Rrs5–20 in IOS measurement, which reflected impaired peripheral lung function [28]. Conducting a similar study in the pediatric asthma population could help establish a link between the perception of dyspnea in children with asthma and potential SAD.

### 4.2. IOS in Children (School-Aged and Older)

Multicenter studies in the pediatric population remain scarce. Xiao et al. carried out a study to examine how IOS measurements and FeNO levels vary with asthma control in preschool-aged children. Their goal was to explore whether using both IOS and FeNO together could help better assess asthma control and to find specific thresholds that can distinguish uncontrolled asthma. A total of 171 children aged 3 to 6 were enrolled in the study and underwent a 3-month follow-up. In the study, FeNO was the best tool to predict asthma control, but additional assessment of IOS measurements resulted in an enhancement of predictive accuracy [29]. Another observation from the study was that IOS measurements did not differ significantly between children with well-controlled asthma and healthy controls, suggesting that IOS may be a valuable tool for monitoring treatment response and detecting improvements in lung function. The most important limitation of the study was the absence of spirometry measurements, which prevented direct comparison between IOS and standard lung function tests in preschool children. Further studies need to include a larger group of participants, extended follow-up duration and perform comprehensive multifactorial analyses.

In the study conducted by Leiria Pinto et al., the aim was to investigate the relationship between clinical and functional respiratory parameters and the lack of asthma control in preschool children. A total of 121 children completed the study (107 with asthma, including 53 with uncontrolled asthma). A clinical questionnaire was used and IOS, spirometry and bronchodilator tests were performed. In their study, no significant differences were found in various spirometer parameters in predicting the degree of asthma control. The results of the IOS assessment did not reveal the expected differences between children with asthma and healthy controls, nor between children with controlled and uncontrolled asthma. Interestingly, healthy children exhibited parameter values deviated from the currently used reference standards that were validated for Nordic populations, not for Portuguese populations. These findings underscore the need to develop appropriate population-specific reference values [30].

Lin et al. carried out a prospective cohort study to assess how useful IOS and FeNO are in evaluating asthma control in children. The focus was on detecting SAD and airway inflammation. The study population consisted of 560 asthmatic children and 140 healthy controls aged 6 to 12 years. The test performed included FeNO, spirometry, IOS, bronchodilator response, total Immunoglobulin E (IgE) and Childhood Asthma Control Test (C-ACT). Since there were no established IOS reference values for the studied population, the researchers used raw IOS data rather than predicted percentages. Spirometry served as the reference method for diagnosing asthma. The potential of IOS to distinguish between controlled and uncontrolled asthma was investigated. The study found that IOS parameters with FeNO and spirometry results helped differentiate between uncontrolled asthma, controlled asthma and partially controlled asthma in children. Children with uncontrolled asthma showed more signs of SAD, stronger responses to bronchodilators and higher FeNO levels, suggesting more inflammation. The combination of IOS and FeNO improved the ability to identify uncontrolled asthma, with a specificity greater than 85%. Among IOS markers, Rrs5–20 was especially useful, showing a strong link with both asthma diagnosis and level of control. The study emphasizes the clinical relevance of SAD in uncontrolled pediatric asthma [31].

Nieto et al. explored the impact of a 4-week montelukast treatment on IOS parameters in pediatric asthma patients aged over 5 years with mild persistent asthma. The obtained results were compared with those of a similar group that did not receive the treatment. The study demonstrated a reduction in airway resistance in pediatric asthma patients who were given oral montelukast [32]. IOS could potentially help to assess the treatment response and determine the appropriate indications for administering leukotriene receptor antagonists, potentially in those cases where SAD is identified and an inhaled corticosteroid formulation limits its deposition in the peripheral area of the lung. Accordingly, there is a need to review the effect of extra-fine formulations of inhaled corticosteroids and other inhalant drugs like bronchodilators. Current asthma guidelines do not adequately address the needs of patients who present with the small airways asthma phenotype [33].

A study conducted by Cottini et al. on asthmatic patients who present with SAD, defined by IOS measurements, aims to recognize potential predictors of peripheral airways impairment. In their study, the prevalence of SAD was estimated at 62% among a cohort of 400 unselected asthmatic patients and this phenotype was correlated to risk factors such as overweight, exercise-induced asthma, nocturnal awakenings due to asthma, cigarette smoking and advanced age [34]. Obesity is recognized to be a risk factor for asthma and a determinant that can affect asthma severity and progression [35]. Studies on obesity and asthma in pediatric asthma patients revealed that childhood obesity is correlated to airway dysanapsis, representing the disproportion between the growth of the lung parenchyma and the cross-sectional diameter of the airways. Concerning lung function assessment, it has been established that dysanapsis is associated with increased lung volumes, reduced airflow and increased morbidity in obese pediatric patients with asthma [36].

The impact of obesity on IOS parameters in asthma, other respiratory disorders and non-respiratory conditions was investigated by Ringbaek et al. Results of their study revealed that obesity influenced IOS outcomes among all diagnostic cohorts [37]. Evidence is limited regarding the effect on oscillometric parameters in overweight and obese asthmatic children. According to one of the most recent studies conducted on obese preschool children aged 3 to 7 years, they have increased peripheral and total lung resistance with higher IOS-derived values of R5 and Rrs5–20 in comparison to the control group. The authors concluded that preschool obese children present with diminished respiratory function [38].

### 4.3. IOS in Preschoolers and Infants

The ERS recommends that IOS measurement may be reliably conducted starting at the age of 2 years [39]. There were studies on IOS utility during infancy, but children in the first months of life are habitual nose-breathers, so some modifications of the method were required, such as the use of a face mask, which in consequence influenced the respiratory resistance. The benefit of the study is the development of knowledge about the pathophysiology of respiratory diseases associated with wheezing in infants, but to date without any specific impact on the management of asthma in infants [40].

Waves that are superimposed on patients’ breathing do not change patients’ breathing patterns, and the results reflect the state of the airway that is not affected by the diagnostic procedure; therefore, it does not hinder breathing in a patient with current dyspnea caused by asthma exacerbation [14]. Lack of breathing effort makes this technique applicable in patients with acute infectious respiratory disease, aligning with the overall clinical presentation of childhood asthma. Consequently, a study of the pediatric asthma population and early identification of SAD generates the question of therapeutic decision and early pharmacological intervention. One of the questions is the comparison of the effects of different therapies: systemic therapies with leukotriene receptor antagonists in children in comparison to inhaled corticosteroid therapy.

Research studies are needed to explain if changes in oscillometry-derived parameters in preschoolers may be useful in the detection of acute obstruction and identification of recurrent wheezers from healthy controls. The application in distinguishing asthmatic children in preschool age from healthy children is not clear. Hellinckx et al. were among the first to investigate baseline lung function and bronchodilator response using the IOS technique in asthmatic children and healthy controls aged 2.7–6.6 years old. They proposed a 40% reduction in the predicted value of Rrs5 as a cutoff for a positive bronchodilator response. However, in their study, there were no differences in baseline lung function between asthmatics and healthy controls. The possible explanations are that measurements were taken outside of the exacerbation period, the severity of asthma was not evaluated and half of the asthmatics were treated [10]. Hence, studies are needed on precisely defined, homogenous patient groups, with regard to the oscillometric-derived outcomes in steroid-naïve patients and treated patients over time.

Currently, the largest application of IOS is in the diagnosis of asthma. IOS is used to evaluate bronchodilator response, and specific bronchodilator cutoffs for different oscillometric parameters have been proposed. According to the recommendations of the ERS Technical Standards for Respiratory Oscillometry, bronchodilator response cutoffs are 40% reduction for Rrs5 and 50% increase for Xrs5 in children.

### 4.4. Future Perspectives

IOS is perceived as a method suitable to perform bronchodilator tests and bronchial challenge tests in preschool children; however, standardization of testing protocols is indicated as another future need to be fulfilled. The increasing range of commercially available diagnostic equipment generates difficulties in comparisons of outcomes. A significant limitation that must be acknowledged is the absence of standardized requirements for producers to ensure consistent measurements across different devices. Lack of clear standards for manufacturers makes it difficult to establish normative values for IOS. In contrast, such reference values exist for spirometry and are universally applicable to all individuals based on age, height and sex [41,42]. Multicenter randomized studies based on the same devices as well as unified research protocols and data reporting formats are needed. Comparable equipment and unified protocols should be used in studies before IOS becomes an evidence-based position in asthma management by specialists.

Biological therapies are increasingly used to target specific asthma phenotypes characterized by distinct inflammatory pathways. IOS can aid in phenotyping by detecting SAD, which is often associated with more severe or difficult-to-control asthma. Moreover, the use of extra-fine-particle inhaled corticosteroids can effectively reach the peripheral airways affected in SAD, complementing biologic treatments and improving overall disease management [43].

Spirometric flow-volume loops are helpful in detecting intrathoracic or extrathoracic upper airway obstruction and could be used alongside with IOS when proximal airway disease is suspected [5].

The practical use of IOS in asthmatic patients has been recently proposed, and indications for assessing SAD by IOS have been outlined. These include asthma diagnosis, evaluation of symptom control and assessment of treatment response [5]. The authors emphasized the particular value of IOS in patients with a confirmed diagnosis of asthma, which remained uncontrolled despite proper inhaler technique and good adherence to the therapy. It is recommended to broaden the diagnostic evaluation by including IOS next to other established diagnostic tools, such as spirometry and FeNO. The proposed clinical algorithm guides further management based on the results of IOS. SAD is indicated by elevated values of Rrs5, Xrs5 and AX and an elevated difference between Rrs5 and Rrs20. Rrs20 typically remains within normal limits. Management of SAD may involve either stepping up the dose of inhaled corticosteroids or switching to an extra-fine-particle inhaler to decrease airway inflammation. Repeat IOS testing would potentially help to assess the effectiveness of the implemented treatment. If IOS findings do not support a diagnosis of SAD, further phenotyping using FeNO, peripheral blood eosinophil count and total IgE may provide valuable insights into the underlying phenotype and guide treatment decisions.

IOS has the potential to support asthma phenotyping in children and should be considered as part of a comprehensive diagnostic approach. In preschool-aged children as well as in older children during asthma exacerbations—when forced respiratory maneuvers are difficult to perform—IOS and post-bronchodilator IOS response may be valuable diagnostic tools. However, additional studies are needed to clarify if a persistent bronchodilator response detected by IOS can serve as an indicator of a higher risk for poor asthma control in the future.

In summary, these future directions, including standardized IOS protocols, integration with other diagnostic tools and personalized therapeutic strategies, have the potential to improve early detection, monitoring and management of SAD in both pediatric and adult asthma populations. Table 2 presents the original publications on the use of IOS.

## 5. Conclusions

Ongoing inflammation in the peripheral airways has been increasingly recognized as an important contributor to poor asthma control. SAD is considered a treatable trait in asthma, making it an important focus for improving asthma control and reducing the risk of exacerbations. When spirometry results are within normal limits, further evaluation may consider the possibility of unrecognized SAD, alongside relevant comorbid conditions and lack of adherence to prescribed therapy. Targeting SAD allows for more precise management and personalized care for patients. Currently, IOS could potentially be integrated into clinical practice, starting with the establishment of comprehensive reference values.

Given the limited evidence regarding SAD in pediatric asthma, there is a clear need for multicenter research to explore the clinical utility of IOS. As the evidence base grows, forthcoming pediatric asthma guidelines may potentially integrate the assessment of SAD as a component of routine management.

Spirometry is still the gold standard, but routine asthma management is gradually evolving toward a more comprehensive functional lung assessment. This involves using complementary tools to investigate not only the large airways, with IOS offering the advantage of applicability from childhood to the elderly.

**Figure 1:**
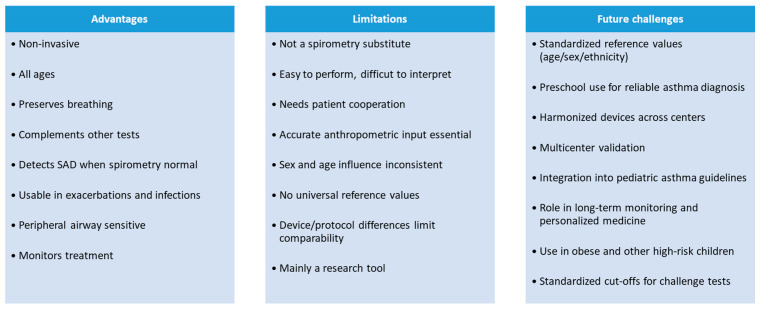
The essential pros, cons and anticipated difficulties of IOS.

Overall, integrating IOS into pediatric asthma management holds considerable promise. Further standardized research is needed to fully define its role in clinical practice and to optimize management strategies for children with SAD. The key advantages, limitations and future challenges of IOS are summarized in the final box (Figure 1).

## Figures and Tables

**Table 1:** Potential methodological errors and measurement artifacts impacting the accuracy of the results.

Error related to height, sex
Inadequate measurement duration for each recording
Insufficient number of repetitions
Absence of nose clip during measurement
Lack of manual cheek stabilization
Incorrect head and body positioning
Saliva swallowing interfering with data acquisition
Breath-holds during testing
Glottic closure events
Air leak around the mouth

**Table 2:** Summary of studies included in the narrative review.

Citation, Author, Year	What Was the Study About?	Study Population
Bar-Yishay E et al., 2009 [6]	- Detection of bronchodilatation in children - Comparison to spirometry	Preschool children
Postma DS et al., 2019 [7]	- Comprehensive assessment of asthma (spirometry, body plethysmography, IOS, multiple breath nitrogen washout, computed tomography in selected participants, questionnaires) - Exploring the relevance and extent of SAD in asthma	Adults with and without asthma
Cauberghs M et al., 1989 [9]	- Impact of upper airway shunt on respiratory impedance assessment	Healthy adults and children and patients with obstructive lung disease
Hellinckx J et al., 1988 [10]	- Evaluation of baseline lung function and bronchodilator response	Preschool healthy and asthmatic children
Calogero C et al., 2013 [11]	- Development of reference values for respiratory impedance	Healthy preschool- and school -aged children
Frei J et al., 2005 [12]	- Development of reference equations - Assessment of changes in IOS parameters in relation to anthropometric measures	Healthy preschool- and school-aged children
Dencker M et al., 2006 [13]	- The extension of the reference values for IOS variables - Assessment of changes in IOS parameters in relation to anthropometric measures	Preschool and school-aged children
Cottini M et al., 2021 [25]	- Evaluation of IOS-defined SAD across treatment steps	Adults with asthma
Takeda T et al., 2010 [28]	- Using IOS to evaluate the relationship between central and peripheral airway function and clinical parameters such as health status, dyspnea and asthma control	Adult patients with asthma
Xiao J et al., 2024 [29]	- Differences in IOS and FeNO in relation to asthma control - Predictive value of IOS combined with FeNO for uncontrolled asthma	Preschool children with asthma and healthy controls
Leiria-Pinto P et al., 2021 [30]	- The association between clinical and functional parameters and the lack of asthma control in preschool children	Preschool children with asthma and healthy controls
Lin LM et al., 2022 [31]	- The role of IOS and FeNO for assessing childhood asthma control in terms of SAD and airway inflammation - Comprehensive evaluation of pediatric asthma using FeNO, spirometry, IOS, bronchodilator testing, total IgE measurement and C-ACT	Asthmatic children and healthy participants.
Nieto A et al., 2006 [32]	- Evaluation of the effect of oral montelukast on airway resistance	Children with asthma and healthy controls
Cottini M et al., 2020 [34]	- Determination of predictors of SAD	Adults with asthma
Ringbaek T et al., 2025 [37]	- Analyzing predictors of respiratory dysfunction across Body Mass Index groups	Adult patients with asthma and/or lung-related symptoms
Klubdaeng A et al., 2025 [38]	- Identification of obesity indices predictive of impaired lung function	Obese preschool children and healthy controls
Hall GL et al., 2002 [40]	- Examining airway resistance changes in nasally breathing infants	Infants
Chan R et al., 2024 [43]	- Evaluation of the effect of biological anti-asthmatic therapy on small airway mechanics	Adult with asthma

## Data Availability

Not applicable.
